# Nuclear receptor subfamily 4 group A member 1 promotes myocardial ischemia/reperfusion injury through inducing mitochondrial fission factor-mediated mitochondrial fragmentation and inhibiting FUN14 domain containing 1-depedent mitophagy

**DOI:** 10.7150/ijbs.95853

**Published:** 2024-08-19

**Authors:** Junyan Wang, Haowen Zhuang, Lianqun Jia, Xinyong He, Sicheng Zheng, Kangshou Ji, Kang Xie, Tong Ying, Ying Zhang, Chun Li, Xing Chang

**Affiliations:** 1 School of Pharmaceutical Sciences, Guangzhou University of Chinese Medicine, Guangzhou, Guangdong, 510006, China.; 2 Liaoning University of Traditional Chinese Medicine, Shenyang, Liaoning, 110032, China.; 3 Senior Department of Cardiology, The Sixth Medical Center of People's Liberation Army General Hospital, Beijing 100048 Beijing, China.; 4 Guang'anmen Hospital, China Academy of Chinese Medical Sciences, Beijing, 100053, China.; 5 State Key Laboratory of Dampness Syndrome of Chinese Medicine, The Second Affiliated Hospital of Guangzhou University of Chinese Medicine, Guangzhou, Guangdong, 510006, China.; 6 Xianning Medical College, Hubei University of Science & Technology, Xianning 437000, China.

**Keywords:** NR4A1, Mff, FUNDC1, pan-apoptosis, myocardial ischemia-reperfusion injury

## Abstract

This study investigated the mechanism by which NR4A1 regulates mitochondrial fission factor (Mff)-related mitochondrial fission and FUN14 domain 1 (FUNDC1)-mediated mitophagy following cardiac ischemia-reperfusion injury(I/R). Our findings showed that the damage regulation was positively correlated with the pathological fission and pan-apoptosis of myocardial cell mitochondria. Compared with wild-type mice (WT), NR4A1-knockout mice exhibited resistance to myocardial ischemia-reperfusion injury and mitochondrial pathological fission, characterized by mitophagy activation. Results showed that ischemia-reperfusion injury increased NR4A1 expression level, activating mitochondrial fission mediated by Mff and restoring the mitophagy phenotype mediated by FUNDC1. The inactivation of FUNDC1 phosphorylation could not mediate the normalization of mitophagy in a timely manner, leading to an excessive stress response of unfolded mitochondrial proteins and an imbalance in mitochondrial homeostasis. This process disrupted the normalization of the mitochondrial quality control network, leading to accumulation of damaged mitochondria and the activation of pan-apoptotic programs. Our data indicate that NR4A1 is a novel and critical target in myocardial I/R injury that exertsand negative regulatory effects by activating Mff-mediated mito-fission and inhibiting FUNDC1-mediated mitophagy. Targeting the crosstalk balance between NR4A1-Mff-FUNDC1 is a potential approach for treating I/R.

## Introduction

Myocardial ischemia/reperfusion (I/R), a common form of ischemic heart disease, is difficult to overcome due to the lack of ideal targeted drugs 1, necessitating the determination of the pathological underlying myocardial I/R injury to facilitate the development of new therapeutic drugs. Inflammatory damage, redox imbalance, metabolic reprogramming, energy metabolism dysfunction, and mitochondrial DNA damage are important pathological mechanisms underlying I/R injury, of which mitochondrial dysfunction is a key mechanism 2. Ischemic and hypoxic stress can activate endogenous mitochondrial quality control (MQC) mechanisms, primarily mitochondrial fission and mitophagy. Under physiological conditions, the pathological fission of mitochondria followed by mitophagy selectively clears damaged or non-functional mitochondria, which cooperates with the normalization of the unfolded mitochondrial protein response (UPRmt) to maintain mitochondrial protein homeostasis 3; 4. Finally, new organelles are produced via mitochondrial biosynthesis.

Mitochondrial dynamics include fission and fusion 5. Excessive mito-fission plays a dominant role in myocardial injury. In hearts with I/R, the level of Drp1 increases, whereas that of Mfn2 and Opa1 is downregulated, thereby causing the release of cyto-c and inducing apoptosis 6; 7. Conversely, stimulating mitophagy and normalizing the FUNDC1 pathway can protect against injury by blocking the mitochondrial pathway-induced apoptosis. Moreover, in mice with I/R injury, mitochondrial fission factor (Mff) is significantly increased, whereas mice with Mff gene knockout show a significant reduction in the infarction area and recovery of cardiac ejection function 8. Furthermore, defects in Mff can block the oligomerization of VDAC1 and excessive opening of mitochondrial permeability transition pores, alleviate damage to respiratory chain complex, and inhibit the activation of oxidative stress and apoptosis pathways 9. Coincidentally, I/R-induced myocardial ischemic injury promotes the excessive activation of JNK, thereby mediating the activation of the Mff pathway. Mff overexpression promotes pathological mitochondrial fission and activates the mitochondrial apoptotic pathway. However, transgenic DUSP1 can block the excessive activation of Mff, reduce severe mitochondrial fission, and provide a survival advantage in myocardial ischemic injury after I/R stress 10. Based on the above findings, we hypothesize that mitophagy and mitochondrial fission are important involved in maintaining mitochondrial homeostasis and are potential therapeutic targets against myocardial I/R injury. However, the upstream regulatory proteins require further investigation.

The nuclear receptor family comprises over 150 members that establish connections between stress stimuli and gene expression and participate in regulating the physiological and pathological functions of multiple organs. Nuclear receptors have multiple biological regulatory effects on cell proliferation, differentiation, apoptosis, metabolism, and evolution. The orphan nuclear receptor neurotrophic factor-induced gene B family includes three members, NR4A1, NR4A2, and NR4A3, that play synergistic regulatory roles in energy metabolism 11; 12; 13. NR4A1, which belongs to the nuclear hormone receptor superfamily, can quickly sense and respond to changes in the extra-environment 14; 15. Various physiological signals and physical stimuli can induce expression of this receptor. NR4A1 is expressed in the muscles, liver, prostate, and nervous system of various rodents, where it plays a dual role in mediating cell proliferation, differentiation, and apoptosis 16. NR4A1 has been shown to exacerbate myocardial I/R injury and promote changes in mitochondrial morphology and structure. Inhibition of NR4A1 can suppress oxidative stress damage and mitochondrial homeostasis disorders, thereby reducing myocardial I/R injury. Moreover, the specific gene knockout of NR4A1 can mediate the upregulation of OPA1 expression, increase mitochondrial fusion, and block the inflammatory response and apoptosis of the mitochondrial pathway 17. NR4A1 promotes the activation of Drp1 and inhibits the transcription of the mitophagy regulatory protein Bnip3, leading to mitochondrial fission overactivation mediated by Drp1 and mitophagy dysfunction mediated by BNIP3 18. NR4A1 also exacerbates liver cell apoptosis, leading to liver dysfunction and chronic metabolic damage, by regulating abnormal mitochondrial fission. Notably, the pathological regulatory mechanism of NR4A1 in cardiogenic ischemic injury has been confirmed 19; 20.

Using *in vitro*/*in vivo* model, this study elucidated the pathological regulatory mechanism of NR4A1 in I/R injury and identified NR4A1 as a potential key protein in I/R. We also confirmed that the pathological upregulation of NR4A1 in I/R injury mediates Mff-associated mitochondrial fission and FUNDC1-related mitophagy dysfunction. This process results in dysregulation of mitochondrial protein homeostasis and biosynthetic dysfunction, ultimately promoting the activation of the pan-apoptotic pathway in cardiomyocytes.

## Materials and Methods

### Animals

Based on previous studies 19; 21, we utilized NR4A1^CKO^, NR4A1^TG^, FUND C1^CKO^, and FUNDC1^TG^ mice (CKO: Cardiac specific knockout; TG: Transgenosis). The experimental protocol was approved by the Ethics Committee of Guangzhou University of TCM in compliance with the ARRIVE Animal Research Guidelines (Reference Number: 20211207002). Adult male C57BL/6 mice, aged 7-8 weeks, were housed at the Animal Experiment Center of Guangzhou University of TCM. The laboratory conditions were maintained at a temperature of 23-25 °C, with a 12-h light/dark cycle, a humidity level of 55% ± 5%, and provided with approved standard feed and drinking water.

### Myocardial ischemia-reperfusion models

To establish a mouse I/R injury model, C57BL/6 mice were anesthetized with an intraperitoneal injection of pentobarbital at a dose of 50 mg/kg. We confirmed the depth of anesthesia by ensuring no physiological response to hindlimb clamping using hemostatic forceps, following which a hair removal cream was applied to depilate and prepare the chest skin locally. The mice were placed on a small constant temperature operating table set at 35-37 ºC, and a cardiac monitor was installed.

After local disinfection, the intercostal skin was cut, muscles were separated, and pericardium was opened. The heart was gently exteriorized to expose the left anterior descending coronary artery, which was swiftly ligated using a 6-0 sterile silk thread with a sliding knot. Ischemia was confirmed by whitening of the pathological area at the base of the heart. The chest was promptly closed and compressed to prevent pneumothorax, and the muscles and skin were sutured in layers.

This procedure induces myocardial ischemia in the anterior wall of the LV. After 45 min of ischemia, a second thoracotomy was performed to loosen the ligature, thereby restoring the blood flow to the left outer branch of the coronary artery and myocardial tissue. The chest was closed and compressed to prevent a pneumothorax.

The sham surgery group underwent the same procedures of anesthesia, thoracotomy, and thread insertion without inducing myocardial ischemia or chest closure. Postoperatively, mice received intramuscular injections of penicillin (18,000 U/kg) for three days to prevent infection 7; 9; 22

### Cardiomyocyte culture

This study used an *in vitro* H/R model of myocardial cells. Based on our previous methods 7; 9; 22, cardiomyocytes were isolated from wild-type (WT) and NR4A1^CKO^/NR4A1^TG^ mice and subsequently used to induce an *in vitro* model of H/R injury. Cells were cultured for 6 h under standard conditions to induce reoxygenation damage. HL-1 cells were transfected with adenovirus I/R for 48 h, with interventions of either 100 mM ethanol or PBS.

### Histology, immunohistochemistry, and immunofluorescence

Extracted mouse hearts were fixed in 10% formalin, dehydrated, and embedded in paraffin. Subsequently, 5 µm thick heart sections were stained with H&E to evaluate the morphological characteristics post-ischemia-reperfusion. Masson staining was used to observe myocardial fibrosis and the extent of injury. Pathological changes in tissues were observed and photographed using an optical microscope. Semi-quantitative analysis of H&E- and Masson-stained myocardial injury areas was performed using the Image Pro software, and the results were expressed as a percentage of the total examined myocardial area.

For immunofluorescence, cell preparations were fixed with 4% paraformaldehyde at 4 °C for 15 min, followed by overnight incubation with the primary antibody at 4 °C. The samples were washed thrice with TBS (5 min per wash) and incubated with a secondary antibody (IgG-Alexa Fluor 555, 1:500; #4409; Cell Signaling Technology, Danvers, MA, USA). Samples were scanned and photographed using an immunofluorescence microscope 7.

### Cardiomyocyte viability and degree of mitochondrial permeability pore transition and opening

The survival rate of cultured neonatal cardiomyocytes was assessed using CCK-8. The mPTP opening rate was evaluated using as previously described. Briefly, control and ethanol-treated cardiomyocytes were washed with PBS and incubated with 10 nM TMRE for 30 min. TMRE fluorescence intensity was recorded every 15 s using a Nikon confocal microscope. A curve describing the variation in fluorescence intensity over time was obtained and the TMRE fluorescence quenching time was normalized to that of the control group to evaluate the mPTP openness rate.

### ELISA

The levels of interleukin (IL)-10, IL-17, TNF-α, MMP9, Complex-I, Complex-III, and Complex-V were measured using ELISA kits (MyBioSource, CA, USA) according to the instructions.

### RT-PCR

Total RNA was isolated from the heart tissues of different experimental groups using TRIzol reagent (Invitrogen). The homogenate was vigorously shaken and incubated on ice for 15 min with aliquots of 0.2 ml chloroform per 1 ml of homogenate. Transfer the mixed sample to a new test tube and add the same volume of isopropanol. The sample was incubated at 4 °C for 10 min and centrifuged at 12,000 rpm for 10 min at 4 °C. The supernatant was removed, and the pellet was washed with 75% ethanol and centrifuged at 8,000 rpm for 8 min at 4 °C. RNA (2 µg) was reverse transcribed using M-MLV reverse transcriptase (Invitrogen), and the resulting cDNA was stored at -80 °C until use. qRT-PCR was performed using SYBR Select Master Mix (Roche, Basel, Switzerland) and the ViiA 7 real-time PCR system (Applied Biosystems, Waltham, Massachusetts, USA).

### Western blotting and co-immunoprecipitation

Briefly, mouse heart tissue and cell samples were lysed using RIPA buffer containing a mixture of protease inhibitors. Equal amounts of proteins were transferred to an SDS-PAGE gel, separated by electrophoresis, and transferred onto a PVDF membrane (IPVH00010, Merck Millipore, Burlington, MA, USA). The protein samples were added dropwise into the wells and supplemented with 20 µl of standard diluent. Subsequently, 200 µl of BCA working solution was added to each sample and incubated at 37 °C for 30 min. The protein sample buffer (loading buffer) was heated in a metal bath at 95 °C for 5 min to achieve protein denaturation and ensure complete protein imprinting.

Prior to the experiment, the PVDF membrane was transferred to a 0.45 µm current with a constant current aperture of 300 mA for 180 min, immersed in 5% BSA-TBST solution, and incubated for 1 h at room temperature. For primary antibody incubation, the membrane was incubated overnight at 4 °C with the appropriate dilutions. The membrane was washed thrice with TBST, 10 min each wash.

For secondary antibody incubation, the membrane was treated with 5% BSA-TBST-diluted secondary antibodies (goat anti-rabbit IgG (H+L) HRP 1:10,000 and goat anti-rabbit IgG (H+L) HRP 1:10,000) and incubated for 60 min at room temperature. The membrane was then washed thrice with TBST for 10 min each wash. ECL was applied to the membrane surface for 3 min. The membrane was subsequently exposed for 10 s to 5 min, based on the light intensity, developed for 2 min, and fixed.

After scanning the images, semi-quantitative data analysis was performed using the ImageJ software and a gel imaging system. The primary antibodies used in this study were: anti-FUNDC1 (ab224722), anti-Beclin-1 (ab62557), anti-ATG5 (ab109490), anti-DRP1 (ab184247), PGC1α (ab77210), anti-Fis-1 (ab156865), anti-Mff (ab129075), anti-Opa1 (ab157457), anti-Mfn1 (ab126575), anti-Mfn2 (ab124773), anti-SLC7A11 (ab307601), anti-GPX-4 (ab125066), anti-Caspase-1 (ab138483), anti-Caspase-8 (ab25901), anti-IL-18 (ab207323), anti-NLRP3 (ab263899), anti-AIM2 (ab119791), anti-Pyrin (ab195975), anti-ZBP-1 (ab290736), anti-GSDMD (ab209845), anti-MLKL (ab196436), anti-RIPK3 (ab195117), anti-NR4A1 (ab153914), anti-Nrf-1 (ab175932), anti-PGC1-α (ab191838), anti-Tfam (ab252432), anti-TNF-α (ab205587), and anti-MMP-9 (ab58803).

### NR4A1-related protein molecular docking

Docking simulations were performed using AutoDock Vina v.1.1.2 to investigate the binding modes of NR4A1 and Mff/FUNDC1. Three-dimensional (3D) structures of NR4A1 and Mff/FUNDC1 were obtained from the RCSB Protein Data Bank (www.rcsb.org). Docking input files were generated using AutoDockTools 1.5.6 software package. The default parameters were applied to Vina docking. The best-scoring trends from the Vina docking results were selected for further analysis. Visual analysis of the docking results was conducted using the PyMOL 1.7.6 software (www.pymol.org).

### Statistical analyses

The results are presented as mean ± standard error of the mean (SEM). Comparisons between two groups were performed using either the parametric *t*-test or the non-parametric Mann-Whitney test, while comparison between more than two groups was conducted using a one-way analysis of variance (ANOVA) followed by the Bonferroni post-hoc test. Data were analyzed using GraphPad Prism 9.0. Statistical significance was set at *p* < 0.05

## Results

### NR4A1 mediates cardiac I/R injury and leads to mitochondrial dysfunction

To verify the regulatory role of NR4A1 in I/R-induced myocardial injury, we established a wild-type (WT) mouse model of I/R and performed western blot analysis.

**Figure 1:**
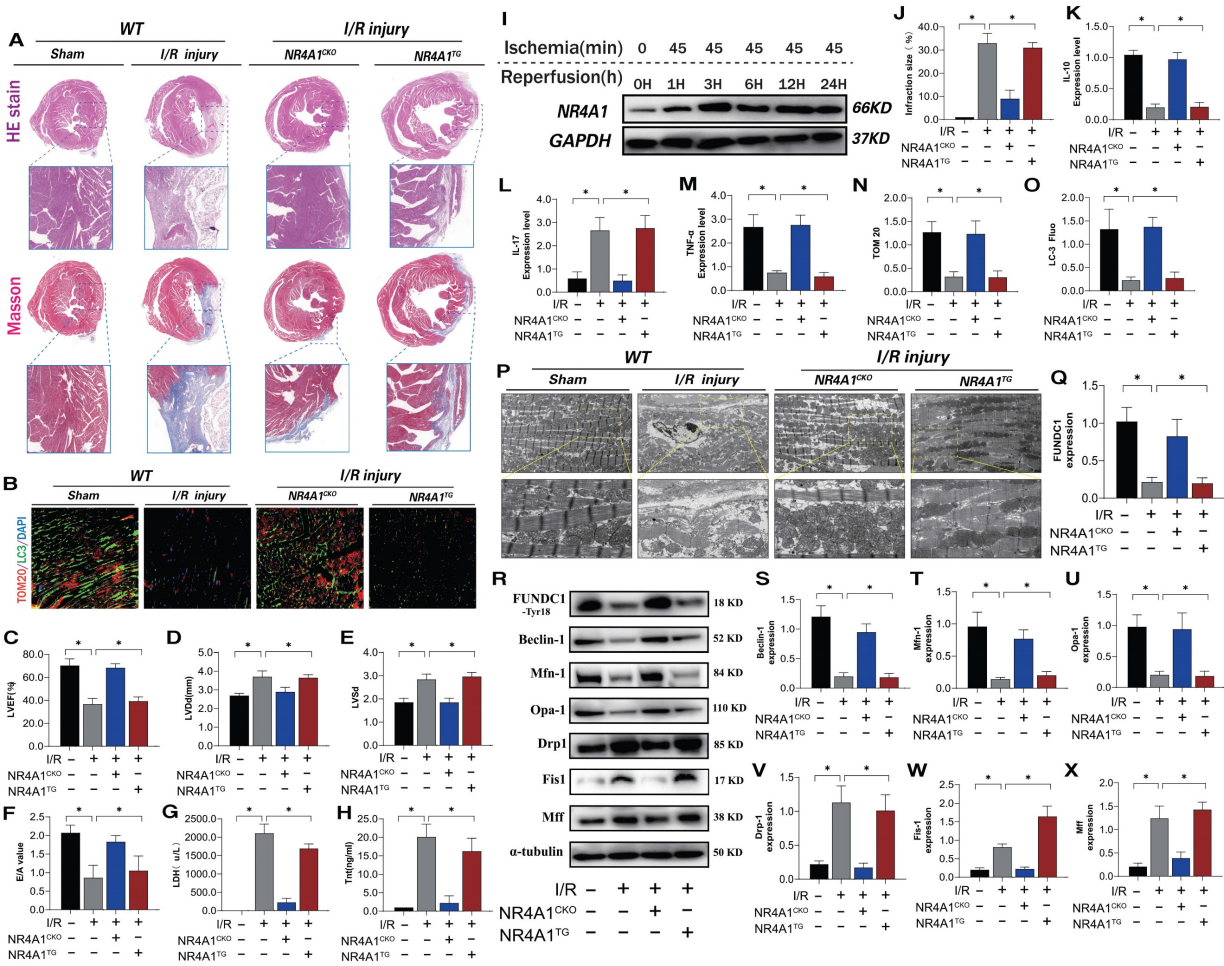
** NR4A1 mediates cardiac I/R injury and leads to myocardial mitochondrial dysfunction.** (A, J) Detection of myocardial ischemic injury area by cardiac pathological staining (HE/Masson); (B, N, O) Immunofluorescence staining and LC3/TOM20 expression level of myocardial tissue); (C-E) Detection of cardiac ejection function (LVEF; LVDd; LVSd; E/A); (G) LDH expression level; (H) Tnt expression level; (I) Western blot analysis of myocardial NR4A1 protein expression in WT mice; (K) The expression levels of anti-inflammatory factor IL-10; (L, M) and inflammatory factor IL-17/TNF-α; (P) Detection of mitochondrial morphology and structural damage in myocardial tissue using transmission electron microscopy; (Q-X) Expression levels of mitochondrial mitophagy related proteins (FUNDC-1/Baclin-1), mitochondrial fusion related proteins (Mfn1/Opa1), and mitochondrial fission related proteins (Drp-1/Fis1/Mff); Experiments were repeated at least three times and the data are shown as mean ± SEM. *P<0.05.

NR4A1 expression increased over time following I/R injury (Figure 1I). To evaluate the causal relationship between NR4A1 and myocardial I/R injury, we conducted pathological injury control experiments in myocardium-specific NR4A1 knockout (NR4A1^CKO^) and NR4A1 transgenic (NR4A1^TG^) mice and found that I/R led to increased myocardial injury markers, structural damage to myocardial fibers, disordered arrangement, swelling, rupture, and abnormal cardiac function (Figure 1A-H, J). This resulted in severe myocardial injury and impaired cardiac ejection function in the control mice, along with exacerbated inflammatory injury and morphological damage to myocardial mitochondria (Figure 1K-M, P). The injury process also decreased mitophagy (Figure 1B, N-O). However, these indicators were significantly reduced in NR4A1^CKO^ mice, whereas NR4A1^TG^ did not ameliorate these pathological changes (Figure 1A-P). These results indicate that NR4A1 is a crucial regulatory protein mediating myocardial I/R injury and that NR4A1^CKO^ protects the mouse myocardium from structural and functional abnormalities induced by I/R (Figure 1K-M,P). Furthermore, mitochondrial function assays revealed that, compared with the control group mice, mitophagy-mediated proteins exhibited low expression following I/R (Figure 1R-X). The level of mitophagy mediated by FUNDC1 significantly decreased following I/R and was accompanied by dysregulated mitochondrial dynamics (Figure 1R-X). This suggests that NR4A1 exacerbates I/R damage by mediating excessive mitochondrial fission and mitophagy dysfunction. Based on these findings, we conducted gene sequencing experiments using NR4A1 genetically modified mice.

### Correlation between NR4A1 mediated cardiac I/R injury and mitochondrial fission and mitophagy

**Figure 2:**
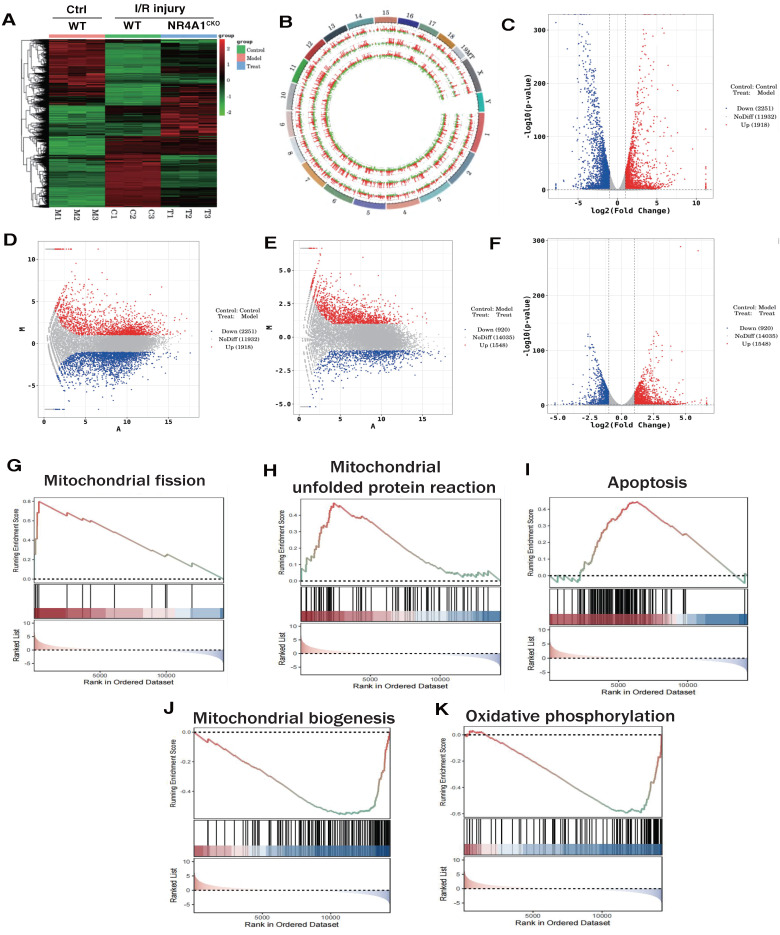
** The correlation between NR4A1 mediated cardiac I/R injury and Mitochondrial.** (A-B). Heatmap and genome-Circos plots of gene expression in the control group, I/R model group, and I/R model group (NR4A1^CKO^); (C-D) Volcano plot and MA plot of differential genes between the control group and the I/R model group; Volcano plot and MA plot of differentially expressed genes between the control group and the NR4A1 knockout I/R model group; (E-F) (G-K) GSEA analysis showed that compared to the control group, the myocardial cells of the I/R model group mice exhibited significant activation of mitochondrial fission, mito UPR, and apoptosis, while mitochondrial biogenesis and OXPHOS were significantly inhibited. Experiments were repeated at least three times and the data are shown as mean ± SEM. *p<0.05.

**Figure 3:**
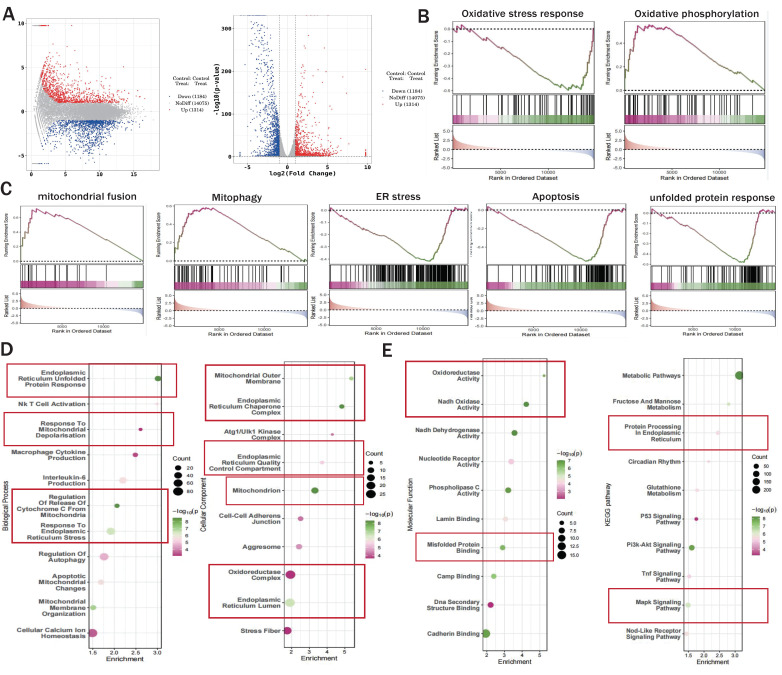
** The correlation between NR4A1 mediated cardiac I/R injury and mitochondrial quality control.** (A) Volcano plot and MA plot of differential genes between I/R model groups; (B-C) GSEA analysis showed that compared to the I/R model group mice, cardiomyocyte specific knockout of NR4A1 can effectively alleviate oxidative stress, ER stress, apoptosis, the unfolded mitochondrial protein response contributes to the restoration of key mitochondrial quality control pathways such as OXPHOS and mitochondrial fusion, the enrichment analysis of Biological Process, Cellular Component, and Molecular Function in (D-E) GO analysis showed that the differentially expressed genes in the NR4A1 knockout group of cardiomyocytes were mainly enriched in mitochondria, endoplasmic reticulum, UPRmt, and other aspects. The KEGG pathway also demonstrates that NR4A1 knockout mainly affects core pathways such as myocardial cell metabolism and endoplasmic reticulum protein processing. Experiments were repeated at least three times and the data are shown as mean ± SEM. *P<0.05.

We preliminarily confirmed that NR4A1-mediated mitochondrial fission and mitophagy dysregulation may be key mechanisms leading to I/R injury. To confirm the correlation between these mechanisms, we constructed an NR4A1 gene-modified mouse I/R model and performed second-generation transcriptome sequencing validation. The results confirmed that the NR4A1-mediated I/R injury mechanism is primarily associated with mito-fission/fusion, mitophagy, UPR^mt^, biosynthesis, oxidative phosphorylation, and apoptosis (Figure 2A-K). Furthermore, NR4A1 mediated the differential expression of genes related to endoplasmic reticulum stress injury and regulation of mitochondrial protein homeostasis (Figure 3A-E). These findings suggesting that mitochondrial dysfunction is the core pathological mechanism underlying I/R injury.

**Figure 4:**
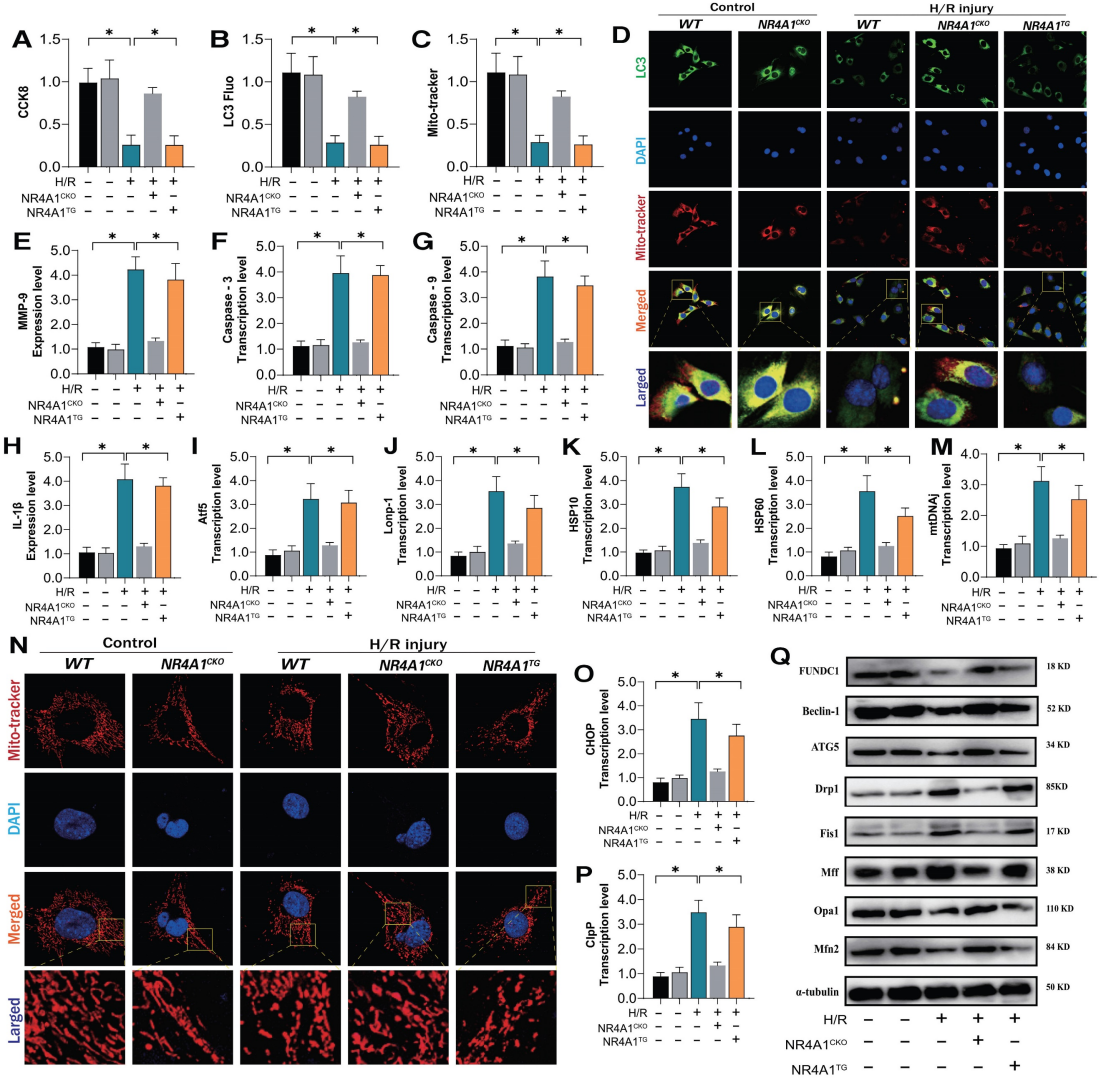
** NR4A1-FUNDC1 mediated mitochondrial fission regulates mitochondrial biosynthesis in cardiomyocytes after I/R injury.** CCK8 detection of myocardial cell activity; (B-D) Fluorescence co localization detection of co localization expression of LC3 and Mito Tracker; (E) Inflammatory cytokine MMP-9 activity; (F-H) Caspase pathway cell apoptosis expression level; (I-M, O-P) UPR^mt^ related gene expression levels (Atf5/HSP10/HSP60/Lonp1/CHOP/mtDNAj/CpPP); (N) Laser confocal detection of mitochondrial morphology and structure (Mito tracker); (Q) The expression levels of mitochondrial mitophagy related proteins (FUNDC-1/Baclin-1), mitochondrial fusion related proteins (Mfn1/Opa1), and mitochondrial fission related proteins (Drp-1/Fis1/Mff) were experimentally repeated at least three times and the data were shown as mean ± SEM. * p<0.05.

To confirm the association between NR4A1-mediated I/R injury and mitochondrial function, we determined whether NR4A1 deficiency could reverse the mitochondrial homeostatic phenotype of myocardial cells following H/R. We evaluated the relevant mechanisms via *in vitro* genetic modifications. First, the expression of mitophagy-related markers and cellular activity were assessed using CCK8 and fluorescence co-localization experiments. The results showed decrease in myocardial cell activity following simulated I/R injury, accompanied by a decrease in the expression of mitophagy markers (Figure 4A-D). Additionally, there was an increase in the transcription levels of genes related to the mitochondrial unfolded protein response (UPR^mt^) and mitochondrial fragmentation (Figure 4I-P). I/R injury further increased mitochondrial fragmentation and mitophagy dysfunction, ultimately leading to the activation of programmed cell death pathways and decreased myocardial cell activity (Figure 4E-H,Q). Treatment with NR4A1 did not affect these pathological manifestations (Figure 4A-Q). However, NR4A1 knockout reversed these phenomena, increased mitophagy levels, and inhibited excessive fission and activation of UPR^mt^ (Figure 4A-Q). This suggests NR4A1 as a key protein causing mitochondrial homeostasis imbalance in myocardial cells after ischemia-reperfusion injury, and that its downstream regulatory genes and mechanisms require further explanation.

### NR4A1 inhibits FUNDC1-mediated mitophagy activation and widespread apoptosis of cardiomyocytes following I/R injury

**Figure 5:**
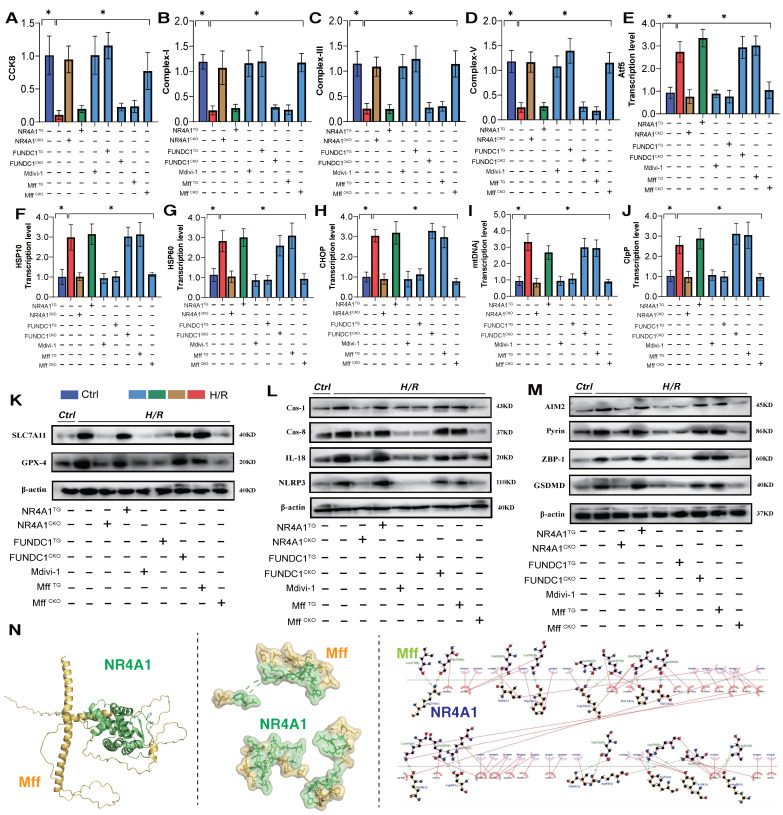
** NR4A1 regulates the activation of pan apoptotic mitochondrial pathway in cardiomyocytes through FUNDC1-Mff.** (A) CCK8 detection of myocardial cell activity; (B-D) Expression level of mitochondrial respiratory chain complex (Complex-I/III/V); (E-J) Transcription levels of UPR^mt^ related genes (Atf5/HSP10/HSP60/Lonp1/CHOP/mtDNAj/CpPP); (K-M) Expression levels of pan apoptotic related proteins (cell pyroptosis, ferroptosis); (N) Molecular docking between Mff and NR4A1; Experiments were repeated at least three times and the data are shown as mean ± SEM. *p<0.05.

NR4A1-mediated mitochondrial homeostasis disorders are closely associated with the pathogenesis of cardiac injury 14. Transcriptomic studies and *in vitro* experiments using NR4A1 gene-modified animal models further confirmed the critical role of mitophagy dysfunction in the programmed cell death of cardiomyocytes. Receptor-dependent mitophagy involves the localization of FUNDC1 mitophagy receptors on the OMM, which directly interact with LC3 to mediate the clearance of damaged or excess mitochondria 23; 24. Following mitochondrial damage, PHB2 and cardiolipin externalize OMM and interact with LC3. Different receptors ensure tissue and stimulus specificity 25. Phosphorylation of FUNDC1 sites enhances their association with LC3, promoting normal activation of mitophagy 26; 27. We investigated the mechanism by which NR4A1 blocks pan-apoptosis by regulating mitophagy via FUNDC1 expression and found that I/R injury led to abnormal activation of the UPR^mt^ and respiratory chain dysfunction in myocardial cells, further mediating the abnormal activation of pan-apoptosis-related proteins (necroptosis, ferroptosis, and pyroptosis) and resulting in decreased myocardial cell activity, and the molecular docking experiment results between Mff and NR4A1 further suggest the interaction between the Mff-NR4A1 (Figure 5A-N). Interestingly, NR4A1 knockout and transgenic treatment with FUNDC1 inhibited excessive stress of the UPR^mt^, restored the respiratory chain complex function, and inhibited the overexpression of pan-apoptosis-related proteins (Figure 5A-N).

Furthermore, the mitochondrial fission inhibitor Medivi-1 exerted the same regulatory effect, with both interventions reversing the overexpression of pan-apoptotic proteins in the mitochondrial pathway, restoring normal mitochondrial function, and maintaining mitochondrial protein homeostasis (Figure 5A-N). This suggests that the pathological mechanism of excessive mitochondrial fission may induce mitophagy dysfunction. To explore the mechanism of mitochondrial fission-mediated apoptosis in cardiomyocytes, we established Mff gene-modification treatments for cardiomyocytes (heart-specific knockout/overexpression) and extracted cardiomyocytes to simulate I/R injury (Figure 5A-N). The results showed that Mff knockout reversed mitochondrial homeostasis imbalance, restored normal mitophagy, inhibited the UPR^mt^, and blocked mitochondrial apoptosis (Figure 5A-N).

FUNDC1^CKO^ and NR4A1/Mff^TG^ gene modification treatments did not alter mitochondrial respiratory chain dysfunction or the excessive stress response to unfolded mitochondrial proteins following I/R injury, nor did they block activation of the pan-apoptotic pathway in cardiomyocytes (Figure 5A-N). These results confirm that NR4A1 regulations of FUNDC1-mediated mitophagy and Mff-related excessive mitochondrial fission are key induction mechanisms leading to mitochondrial dysfunction and pan-apoptosis of mitochondrial pathways, offering targets for protecting myocardial cell mitochondria. Further experiments are required to confirm the regulatory mechanisms of mitochondrial fission.

### NR4A1-FUNDC1 mediated mitochondrial fission regulates mitochondrial biosynthesis following I/R injury

**Figure 6:**
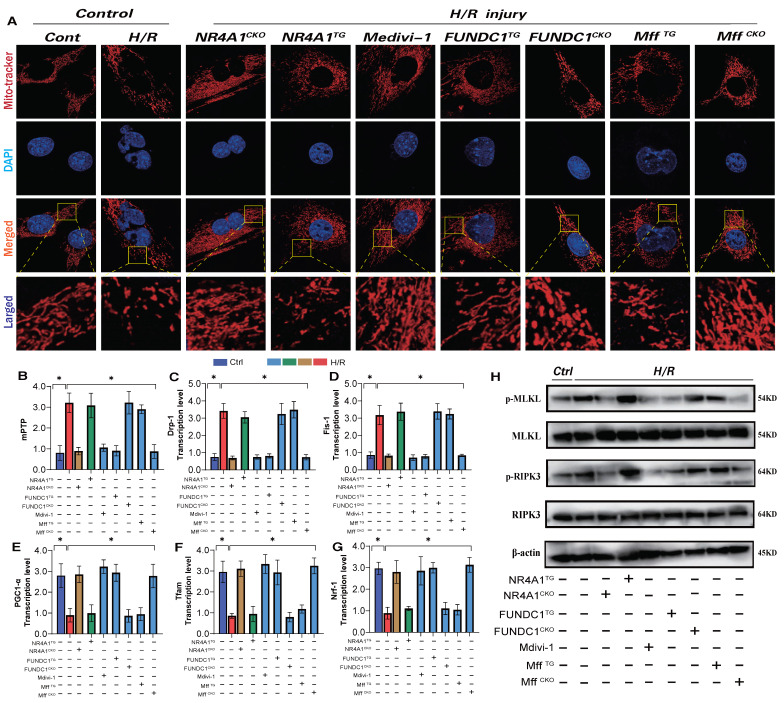
** NR4A1 regulates mitochondrial apoptosis and biosynthesis levels in cardiomyocytes through FUNDC1-Mff.** Mitochondrial morphology and structural integrity testing (Mito-tracker); (B) Detection of mitochondrial membrane permeability transition pore(mPTP) opening degree; (C-D) Detection of mitochondrial fission driving protein expression level (Drp1/Fis1); (E-G) Detection of mitochondrial biosynthesis related gene transcription level (PGC1-α/Tfam/Nrf-1); (H) Detection of mitochondrial pathway necrosis related apoptosis protein phosphorylation level(MLKL/RIPK3); Experiments were repeated at least three times and the data are shown as mean ± SEM (N=three independent cell isolations per group). *p<0.05.

Mitochondrial biosynthesis, the process of regenerating mitochondrial DNA to replace non-functional or damaged mitochondria 28; 29, is primarily regulated by several co-biosynthetic transcription factors, including PGC1α and Nrf-1/-2/Tfam 30. Herein, following I/R injury, we observed a decrease in PGC1α and Nrf-1/-2/Tfam transcription levels, along with the activation of necrotic apoptosis (Figure 6E-G). The dysfunction of mitochondrial biosynthesis mediated by I/R injury further activated the pan-apoptotic mitochondrial pathway. Additionally, there was an increase in mitochondrial fission/fragmentation levels and an abnormal opening of mPTP in cardiomyocytes (Figure 6A-D,H), suggesting that I/R-mediated mitochondrial biosynthesis dysfunction is caused by excessive mitochondrial fission and membrane permeability transition pore dysfunction (Figure 6A-D,H). Intervention with mitochondrial fission inhibitors and NR4A1/Mff gene knockout inhibited excessive mitochondrial fission and fragmentation, blocked the necrotic apoptosis pathway, improved mitochondrial biosynthesis (Figure 6A-H) and inhibited the overexpression of pan-apoptosis-related proteins (Figure 6A-H). Interestingly, FUNDC1 overexpression exerted the same mitochondrial regulatory effect, inhibiting excessive mitochondrial fission and fragmentation and enhancing mitochondrial biosynthesis (Figure 6A-H). However, the FUNDC1^CKO^/NR4A1 ^TG^/Mff ^TG^ gene modification treatments did not alter these effects (Figure 6A-H). Furthermore, mitophagy dysfunction mediated by FUNDC1 under NR4A1 regulation was confirmed to induce mitochondrial fragmentation and MFF-related pathological fission. These results suggests that NR4A1-regulated mitophagy/fission is the core mechanism that mediates myocardial I/R injury.

### NR4A1 disrupts the cytoskeleton homeostasis of myocardial cells following I/R injury by inhibiting FUNDC1-mediated mitophagy

**Figure 7:**
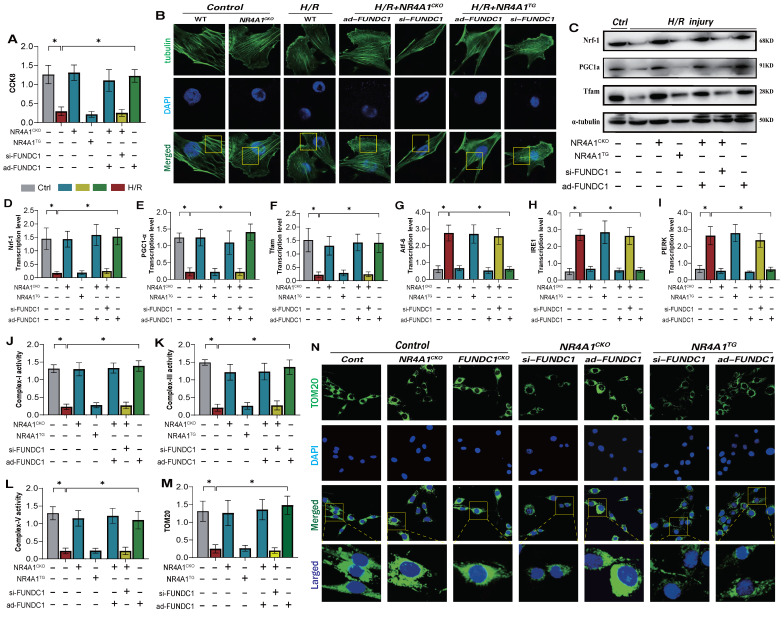
** NR4A1 disrupts the cytoskeleton homeostasis of myocardial cells after I/R injury by inhibiting FUNDC1-mediated mitophagy.** (A) CCK8 detection of myocardial cell activity; (B) Detection of cytoskeleton protein integrity in myocardial cells; (C-F) Expression and transcription levels of mitochondrial biosynthesis related proteins(PGC1-α/Nrf-1/Tfam); (G-I) Transcriptional levels of endoplasmic reticulum stress-related genes(Atf-6/IRE1/PERK); (J-L) Expression level of mitochondrial respiratory chain complex(I/III/V); (M-N) Laser confocal detection of mitochondrial mitophagy marker TOM20 expression level;Experiments were repeated at least three times and the data are shown as mean ± SEM (N=three independent cell isolations per group). *p<0.05.

Under physiological conditions, normal mitophagy is crucial for maintaining intracellular homeostasis and cytoskeletal protein stability in cardiomyocytes 31; 32. This process affects endoplasmic reticulum function and mitochondrial respiratory chain functions, thereby mediating the activation of the cell death program 33; 34. Given that NR4A1 mediates FUNDC1-related mitophagy dysfunction and negatively regulates cardiac mitochondrial homeostasis in mice, we determined whether NR4A1 could directly regulate FUNDC1-mediated mitophagy and affect cellular homeostasis. Confocal and western blot analyses of myocardial cells showed significant damage to cytoskeletal proteins following I/R injury, accompanied by mitochondrial biosynthesis and mitophagy (Figure 7A-F). However, after modifying the si-FUNDC1 gene in primary cardiomyocytes isolated from NR4A1 knockout mice, the cytoskeletal proteins remained damaged, and the reduced level of mitochondrial biosynthesis was not reversed (Figure 7A-F). This was accompanied by mitophagy dysfunction, mitochondrial respiratory chain dysfunction, and severe endoplasmic reticulum stress (Figure 7E-N). Interestingly, modification of the ad-FUNDC1 gene in primary cardiomyocytes isolated from NR4A1 knockout mice did not affect the mitochondrial protective mechanism conferred by NR4A1 knockout. This suggests that NR4A1 exerts a protective effect on cardiac cytoskeletal proteins and mitochondrial DNA by inhibiting FUNDC1-mediated mitophagy (Figure 7A-N).

We generated an I/R injury model using NR4A1 transgenic mice and found that si-FUNDC1 and ad-FUNDC1 gene modifications in primary cardiomyocytes isolated from NR4A1 transgenic mice did not regulate mitophagy/biosynthesis or endoplasmic reticulum stress phenotypes (Figure 7A-N). These findings were further confirming the regulatory effect of NR4A1 on FUNDC1-mediated mitophagy.

### NR4A1 improves myocardial cell activity following I/R by regulating Mff-related mitochondrial fission

Although we have previously confirmed the regulatory mechanism of NR4A1 in FUNDC1-mediated mitophagy, the interactive regulatory mechanism by which NR4A1 promotes Mff-mediated mitochondrial fission remains to be validated. To insights into the mechanism underlying pathological mitochondrial fission disorders induced by I/R stress, we measured the levels of mito-fission and fragmentation in cardiomyocytes and examined the expression levels of mito-fission/fusion-driving proteins.

**Figure 8:**
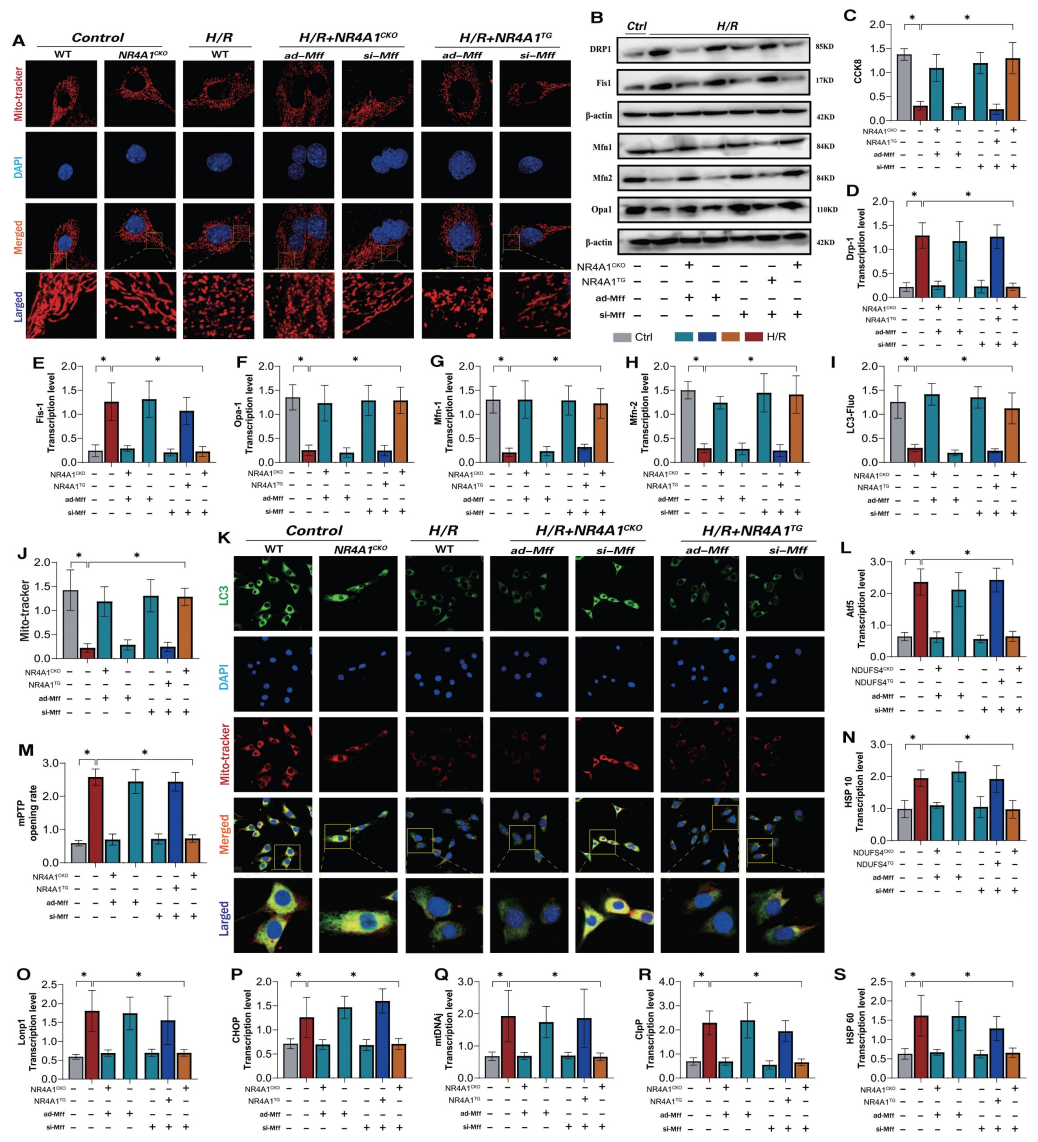
** NR4A1 improves myocardial cell activity after ischemia-reperfusion by regulating Mff related mitochondrial fission.** (A) Mitochondrial morphology and structural integrity testing (Mito-tracker); (B) The expression of mitochondrial fusion related proteins (Mfn1/Opa1) and mitochondrial fission related proteins (Drp-1/Fis1/Mff); (C) CCK8 detection of myocardial cell activity; (D-E) Detection of mitochondrial fission drive protein expression levels (Drp1/Fis1); (F-G) Detection of mitochondrial fusion motor protein expression levels (Opa1/Mfn1/Mfn2); (I-K) Fluorescence co localization detection of co localization expression of LC3 and Mito-Tracker; (L, N-S) Expression of UPR^mt^ related genes (Atf5/HSP10/HSP60/Lonp1/CHOP/mtDNAj/ClpP); Experiments were repeated at least three times and the data are shown as mean ± SEM (N=three independent cell isolations per group). *p<0.05.

I/R led to excessive fission, thereby decreasing myocardial cell activity (Figure 8A-H). Notably, si-Mff intervention and si-Mff treatment based on the NR4A1 knockout inhibited excessive mitochondrial fission and restored mitochondrial fusion and myocardial cell activity (Figure 8A-H). However, Ad-Mff and NR4A1^CKO^\ad-Mff did not reverse these pathological changes (Figure 8A-H). Interestingly, si-Mff and ad-Mff treatments based on the NR4A1 transgenic model (NR4A1^TG^) did not reverse the mitochondrial pathological changes following I/R injury (Figure 8A-H), suggesting that NR4A1 regulates mitochondrial pathological fission through Mff and mediates myocardial cell damage via the mitochondrial pathway. We further explored the effects of NR4A1 regulation on the Mff-mediated abnormalities in mitochondrial fission during mitophagy and found that I/R injury led to the inhibition of mitophagy and excessive stress response to UPR^mt^ (Figure 8I-S). Consistent with previous results, si-Mff intervention and si-Mff treatment based on NR4A1 gene knockout reversed the decrease in mitophagy levels and inhibited the excessive stress response of UPR^mt^ (Figure 8I-S). However, ad-Mff or NR4A1^CKO^ did not reverse these pathological changes, and si-Mff and ad-Mff treatments based on the NR4A1 transgenic model (NR4A1^TG^) did not reverse mitophagy dysfunction or the excessive stress response of unfolded proteins following I/R injury (Figure 8I-S). Furthermore, under I/R injury conditions, NR4A1 regulated Mff-mediated excessive mitochondrial fission, affecting mitophagy and protein homeostasis, thereby exacerbating pathological damage to myocardial cells.

**Figure 9:**
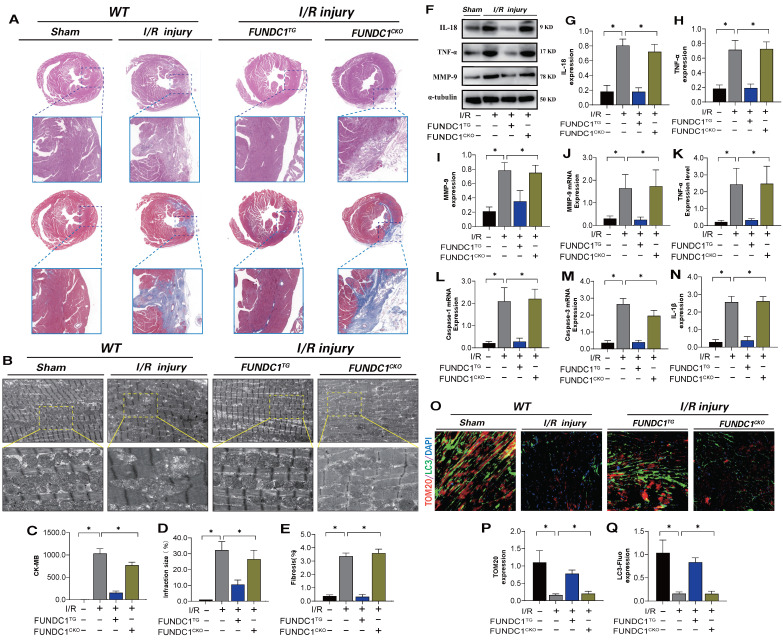
** FUNDC1 improves ischemia-reperfusion myocarditis injury by regulating mitochondrial mitophagy.** (A) Detection of myocardial ischemic injury area by cardiac pathological staining (HE/Masson); (B) Detection of mitochondrial morphology and structural damage in myocardial tissue using transmission electron microscopy; (C) CK-MB expression level detection; (D) Detection of myocardial injury size(%); (E) Fibrosis level detection(%); (F-J) Detection of expression of inflammatory cytokine related proteins and mRNA expression; (K-N) Detection of expression of inflammatory cytokines and mitochondrial pathway apoptosis genes(IL-18/TNF-α/MMP-9) (O-Q) Fluorescence co localization of TOM20/LC3 in myocardial tissue; Experiments were repeated at least three times and the data are shown as mean ± SEM. *P<0.05.

To confirm the mechanism by which FUNDC1-mediated mitochondrial fission leads to myocardial I/R injury, we established FUNDC1 heart-specific knockout I/R models and FUNDC1 transgenic animal I/R models and conducted myocardial injury detection and mitophagy marker analysis. FUNDC1^TG^ reversed myocardial injury after I/R, restored pathological changes in mitochondrial structure and morphology, enhanced mitophagy, and inhibited the inflammatory response in myocardial cells via mitochondrial pathways (Figure 9 A-Q). However, FUNDC1 heart-specific knockout did not reverse the pathological response to myocardial injury (Figure 9 A-Q). *In vivo*, FUNDC1-mediated mitophagy may be the core factor mediating mito-dysfunction and mitochondrial pathway inflammatory damage.

## Discussion

I/R injury remains a major clinical challenge and the leading cause of cardiovascular disease (CVD) incidence and mortality globally 35; 36; 37. Identifying new molecular targets may provide valuable insights into the pathophysiology of clinical I/R injury and guide innovative treatment strategies to reduce the increased mortality and poor prognosis associated with I/R myocardial injury 38; 39. In this study, we determined that NR4A1 was a significant pathological factor that promotedes I/R injury in mice using a genetically modified I/R animal model. Specifically, NR4A1 exacerbated myocarditis-induced injury following I/R and mediated the dysregulation of mitochondrial. Cardiac-specific knockout of NR4A1 reversed myocardial injury and ejection dysfunction caused by I/R, thereby maintaining normal cardiac function. Mechanistically, I/R injury led to mitochondrial homeostasis disorders manifested as mitochondrial respiratory chain dysfunction (decreased expression of mitochondrial respiratory chain complexes) and conditional activation of programmed cell death in the mitochondrial pathway. Notably, I/R-mediated mitochondrial pathological changes can be reversed by heart-specific NR4A1 knockout. Furthermore, NR4A1 directly affected mitochondrial fission/fusion, mitophagy, UPR^mt^, and the activation of mitochondrial biosynthesis via downstream mitochondrial phenotype-regulation mechanisms.

Using genetic modification technology combined with laser confocal microscopy, western blotting, PCR, and other experimental tests, we confirmed that NR4A1 exacerbated myocardial injury by inhibiting mitophagy and activating mito-fission. In summary, these results highlight the crucial roles of NR4A1- FUNDC1 mediated mitophagy and Mff-mediated mito-fission in mouse I/R injury. Therefore, this study explored targeted therapies that inhibit the binding of NR4A1 to FUNDC1/Mff, which may protect against I/R and improve the long-term prognosis of patients.

Abundant evidence supports the critical role of mitochondrial dysfunction in I/R. During myocardial cell damage caused by ischemia and hypoxia, a series of harmful mitochondrial signals are activated, including inflammatory storms, oxidative stress, abnormal energy metabolism, and cellular death processes 40; 41; 42; 43. These processes severely damage the structure of mitochondria, leading to the interaction mechanism of the endoplasmic reticulum and other related organelle damage 44; 45; 46. The mitochondrial quality control (MQC)involves a series of adaptive responses, including mitochondrial fission/fusion, mitophagy, biosynthesis, and UPR^mt^, the activation of which can alleviate mitochondrial damage, mediate the selective degradation of damaged or dysfunctional mitochondria, and ultimately maintain mitochondrial integrity and homeostasis 47; 48; 49. Mitochondrial dynamics (fusion/fission) and mitophagy play central roles in the regulation of the MQC 50; 51.

Studies on mitochondrial fission have found that ROS levels in mouse cardiomyocytes increase to varying degrees following stress stimuli, such as myocardial I/R injury, LPS injury, and diabetes injury 52; 53. Concurrently, an increase in mito-fragmentation or a decrease in mito-length induces cell death. The exacerbation of mito-fragmentation mediated by I/R injury is often accompanied by increased expression of Drp1, Fis1, and Mff 54; 55. Additionally, siRNA treatment of Fis1 or Drp1/Mff inhibitsed mito-fission and myocardial cell damage 56, indicating that mito-fission plays a central role in I/R injury.

Furthermore, the results suggested that I/R injury was accompanied by inhibition of the mitochondrial fusion, as well as a decrease in biosynthesis levels. Further investigation revealed that excessive pathological mitochondrial fission can increase damaged or nonfunctional mitochondria. These mitochondria cannot be engulfed by low-level mitophagy after stress damage, leading to dysregulation of mitochondrial protein homeostasis and mitochondrial energy metabolism, ultimately resulting in the activation of the mitochondrial cell death pathway 57. Research has confirmed that myocardial I/R injury can be attributed to Mff-dependent mito-fission through VDAC1-mediated mPTP opening and cyt-c release involving mitochondrial ROS/cardiolipin, which mediates the exacerbation of myocardial injury following I/R. However, knocking out NR4A1 can block the downstream damage mechanisms mediated by pathological mitochondrial fission. Transgenic NR4A1 also blocked the activation of mitophagy mediated by FUNDC1. These results confirm that NR4A1 regulates Mff-mediated mitochondrial fission, inhibits FUNDC1-mediated mitophagy, and activates the pan-apoptotic pathway 9.

Based on this evidence, we inferred the critical mechanism by which NR4A1 mediates the abnormal expression of mitochondrial MFF and FUNDC1. These findings suggest that maintaining low NR4A1 expression during I/R may be an important protective strategy for maintaining cardiac function. NR4A1 plays a significant regulatory role in the control of cardiac metabolism, mitophagy, inflammatory damage, and mitochondrial biosynthesis. Studies have shown that NR4A1-mediated mitochondrial translocation is the primary target of semaglutide for the regulation of lipid metabolism and NR4A1 is involved in the regulation of cell apoptosis 58. Therefore, the expression of NR4A1 are key factors in mitochondrial dysfunction. Semaglutide regulates the expression of NR4A1, thereby improving ischemic myocardial injury 12. Additionally, one study identified a specific role of NR4A1 in oxidative stress induced by myocardial infarction (MI) in elderly mice, consistent with our experimental results, suggesting that ROS-mediated mitochondrial homeostasis disruption may be an important regulatory mechanism of NR4A1 59.

Previous studies confirmed that an imbalance in mitochondrial homeostasis is the core pathological regulatory phenotype of NR4A1. However, this study validated the mechanism by which NR4A1 activates mitochondrial cell death through Mff-mediated mitochondrial pathological fission.

Mitochondria are dynamic and unstable cellular organelles. Under normal conditions, mitochondrial fission provides sustained ATP for homeostasis of the intracellular environment and participates in the regulation of multiple pathways, such as cytoskeletal stability and cellular energy metabolism, ultimately determining the life cycle of myocardial cells 60; 61. Research has shown that Mff mutant mice die of heart failure due to severe dilated cardiomyopathy. Mff mutations mediate the destruction of mitochondrial morphology and structure and reduce mitochondrial respiratory chain activity, leading to cardiac dysfunction and shortened lifespan in mice. However, regulating the balance of Mfn1/Mff expression can mediate the normalization of mitochondrial fusion and fission, thereby restoring tissue integrity and mitochondrial physiology at the cardiac level 62. Examination of the liver, testes, and cerebellum indicates that the precise balance between fusion and fission is cell-type specific 63. During ischemia-reperfusion injury, Drp1 is significantly upregulated, and its mediated mitochondrial fission leads to the breakdown of mitochondrial protein homeostasis, lipid homeostasis, mitochondrial DNA regeneration, and the ATP supply system 52. Previous studies primarily focused on the role of mitochondrial fission mediated by Drp1 and Fis1 motor proteins in myocardial, kidney, and liver injury 64. Evidence suggesting changes in the expression or upstream regulatory mechanisms of Drp1 receptors is limited, especially Mff. One study found that the increased expression of Mff in coronary microvessels following I/R injury leads to an expansion of infarct size and cardiac dysfunction. The authors showed that Mff upregulation was related to significant microcirculation perfusion defects, blood flow disorders, and structural damage to microvascular endothelial cells, which reversed the pathological mechanism mentioned above. The results also showed that Mff upregulation can lead to excessive division by promoting the translocation of Drp1 to the mitochondria, whereas Mff knockout can inhibit the migration of Drp1 to the mitochondria, thereby suppressing pathological mitochondrial fission 9.

This is consistent with our results, where, under the regulation of NR4A1, Mff can mediate the activation of pathological mitochondrial fission and lead to the dysregulation of mitochondrial protein homeostasis and excessive stress of the UPR^mt^. This process also leads to the breakdown of mitophagy and biosynthesis mechanisms, preventing the normal synthesis of newly formed mitochondrial DNA and ultimately mediating the activation of the mitochondrial apoptotic pathway. Notably, NR4A1-mediated Mff can lead to significant defects in FUNDC1-mediated mitophagy.

FUNDC1 is a mitochondrial outer membrane protein 65. The absence of FUNDC1 significantly inhibits hypoxia-induced mitophagy, indicating that FUNDC1 functions as a mitophagy receptor 33. FUNDC1 is primarily involved in mitophagy triggered by ischemia, hypoxia, and decreased mitochondrial membrane potential. The associated mechanisms are intricately linked to SRC kinase, casein kinase 2 (CK2) kinase, and phosphoglycerate mutase 5 (PGAM5) phosphatase. Additionally, we explored the relationship between these kinases and cardiac injury 5; 6; 7; 66. Following I/R injury, the expression level of mitophagy predominantly depends on the phosphorylation and dephosphorylation status of serine 17 (Ser17), serine 13 (Ser13), and tyrosine 18 (Tyr18) sites on the LI/R motif of FUNDC1. This motif is the principal therapeutic target for innovative drug development 33.

In this study, NR4A1 significantly regulated the phosphorylation of FUNDC1 at the Tyr18 site, thereby mediating ischemia-reperfusion injury and programmed cell death. Our results also indicated that Mff-mediated mitochondrial pathological fission was exacerbated and showed FUNDC1-mediated inhibition of mitophagy. However, heart-specific knockout of NR4A1 significantly reversed this phenomenon by blocking pan-apoptosis and improving myocardial cell activity. This finding suggests NR4A1 as an upstream regulatory target of MFF and FUNDC1 and a critical protein mediating I/R mitochondrial damage. These findings provide novel insights into the roles of NR4A1 in the mitochondrial fission-mitophagy crosstalk associated with I/R injury.

This study has some limitations. We did not conduct differential experimental studies on myocardial cell pan-apoptotic tool drugs in mice with I/R. Our preliminary data showed that necroptosis, mitophagy, and mitochondrial fission inhibitors exerted varying degrees of regulatory effects, both positive and negative, on mitochondrial function in myocardial cells. However, in this study, due to the limitations of animal experiments, we did not include related therapeuticdrug-related grouping. In future studies, we intend to conduct relevant drug experiments. The second limitation is the interference between mitophagy and fission. We could not confirm the interaction mechanism between NR4A1, Mff, and FUNDC1 using immunoprecipitation. In future studies, we intend to conduct interaction mechanistic experiments to confirm the interference mechanism between Mff-mediated mito-fission and FUNDC1-mediated mitophagy. Additionally, transgenic animal systems related to MFF were not included in this study. In future studies, we intend to breed Mff-related genetically modified animal systems to confirm the regulatory role of Mff-mediated mitochondrial fission in ischemic cardiomyopathy. Nevertheless, the findings of this study, combined with evidence that NR4A1 is a potential therapeutic target, provide novel insights into new personalized therapies targeting I/R.

## Supplementary Material

Supplementary figures.
