# Supplementary material for: Nuclear receptor subfamily 4 group A member 1 promotes myocardial ischemia/reperfusion injury through inducing mitochondrial fission factor-mediated mitochondrial fragmentation and inhibiting FUN14 domain containing 1-depedent mitophagy
Source: Int J Biol Sci. 2024 Aug 19;20(11):4458–75. doi: 10.7150/ijbs.95853 (PMC11380451; doi:10.7150/ijbs.95853)

Online Supplementary Figures

Supplemental Figure 1 Increased NR4A1 induced by cardiac IR injury promoted the endothelial death. A. The transcriptional alteration of NR4A1 in WT mice with reperfusion injury. *P < 0.05 *vs.* reperfusion for 0 h group. **B.** Cardiac microvascular endothelial cells (CMECs) were isolated from WT mice, and characterized via CD31 staining and Dil-acetylated LDL intake assay. **C-D.** The proteins were isolated from CMECs after HR injury, and western blots was used to analyze the expression of NR4A1 in vitro. *P < 0.05 *vs.* reoxygenation for 0 h group.

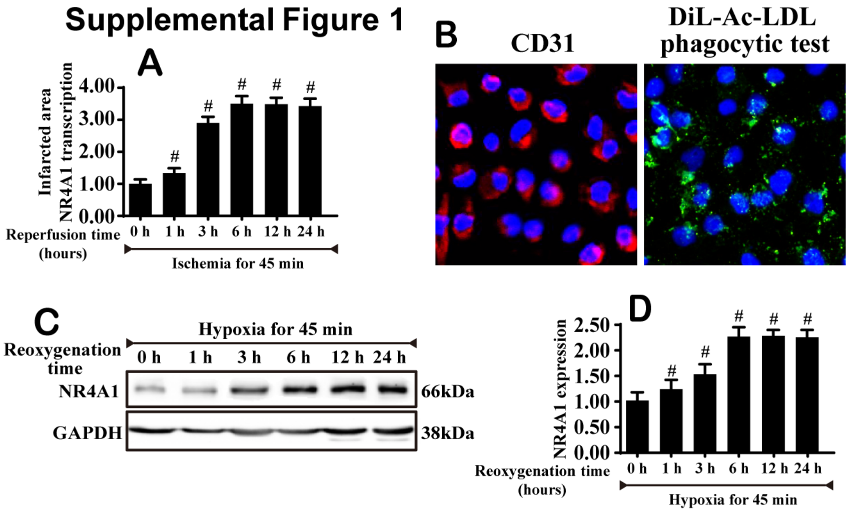

Supplemental Figure 2 Loss of NR4A1 alleviated cardiac damage and reversed cardiac function. A-C. After IR injury, the blood was collected in each group, and the concentration of cardiac damage markers were measured via ELISA assay**. D-F.** The cardiac function was measured via echocardiography. #P < 0.05 *vs.* sham group; *P < 0.05 *vs.* WT+IR group.

Supplemental Figure 2

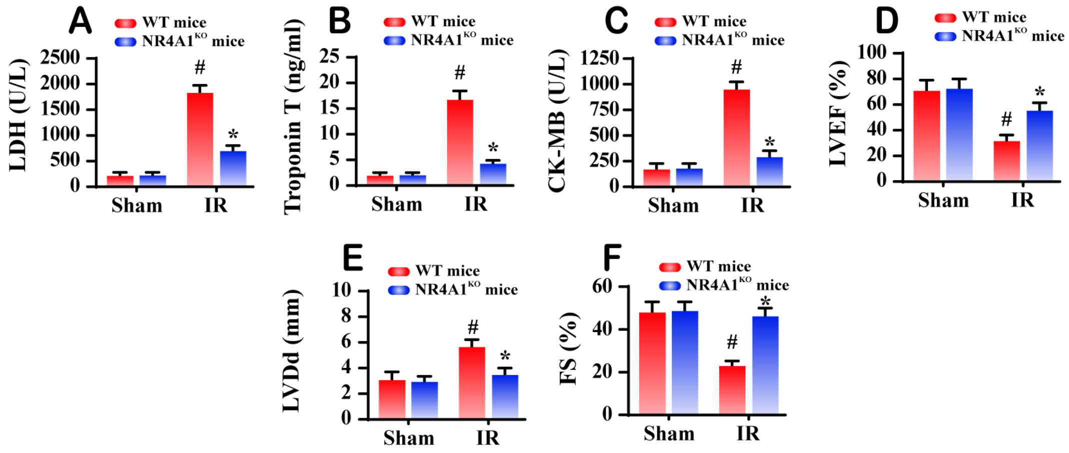

Supplemental Figure 3 Loss of NR4A1 maintained endothelial viability. A-B. CMECs, isolated from WT and NR4A1^{KO} mice, were treated with hypoxia for 45 min and reoxygenation for 6 h in vitro. Then, cellular viability was measured via MTT assay. Besides, caspase-3 activity was measured via ELISA assay. #P < 0.05 *vs.* control group; *P < 0.05 *vs.* WT-cell+HR group.

Supplemental Figure 3

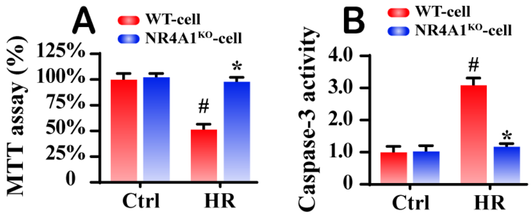

Supplemental Figure 4 Increased NR4A1 induced by cardiac IR injury promoted the endothelial death. A. CMECS, isolated from WT and NR4A1^{KO} mice, were treated with HR injury in vitro. Then, RNA was obtained, and qPCR assay was used to analyze the transcription levels of ET-1. **B.** Fluorescein isothiocyanate (FITC)–dextran clearance

was measured to assess changes in endothelial permeability. FITC-dextran was added on top of the inserts, allowing it to permeate through the cell monolayer. The increased endothelial permeability could retain more FITC-dextran. Thus, the FITC content remaining on the plate after HR injury indicated the extent of permeability of CMECs. **C.** Transendothelial electrical resistance (TER) and permeability examination in CMECs subjected to HR injury. The confluence of CMECs monolayer was assessed as stabilized basal resistance of ~800 Ω. TER increases when endothelial cells adhere and spread out, and decreases when endothelial cells retract or lose adhesion, which is the marker of endothelial barrier function. #$P < 0.05$ *vs.* control group; *$P < 0.05$ *vs.* WT-cell+HR group.

Supplemental Figure 4

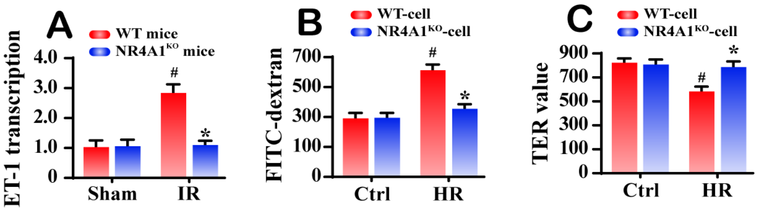

Supplement: Supplementary file 1 — Supplementary figures. [file ijbsv20p4458s1.pdf]
